# CcpA- and Shm2-Pulsed Myeloid Dendritic Cells Induce T-Cell Activation and Enhance the Neutrophilic Oxidative Burst Response to *Aspergillus fumigatus*

**DOI:** 10.3389/fimmu.2021.659752

**Published:** 2021-05-27

**Authors:** Lukas Page, Julia Wallstabe, Jasmin Lother, Maximilian Bauser, Olaf Kniemeyer, Lea Strobel, Vera Voltersen, Janka Teutschbein, Peter Hortschansky, Charles Oliver Morton, Axel A. Brakhage, Max Topp, Hermann Einsele, Sebastian Wurster, Juergen Loeffler

**Affiliations:** ^1^ Department of Internal Medicine II, University Hospital of Wuerzburg, Wuerzburg, Germany; ^2^ Institute for Hygiene & Microbiology, University of Wuerzburg, Wuerzburg, Germany; ^3^ Centre for Image Guided Local Therapies, Otto von Guericke University, Magdeburg, Germany; ^4^ Leibniz-Institute for Natural Products Research and Infection Biology, Hans-Knoell-Institute, Jena, Germany; ^5^ Department of Molecular and Applied Microbiology, Friedrich Schiller University, Jena, Germany; ^6^ School of Science, Western Sydney University, Campbelltown, NSW, Australia; ^7^ Department of Infectious Diseases, The University of Texas MD Anderson Cancer Center, Houston, TX, United States

**Keywords:** *Aspergillus*, antigens, dendritic cells, cytokines, host defense, immunotherapy

## Abstract

*Aspergillus fumigatus* causes life-threatening opportunistic infections in immunocompromised patients. As therapeutic outcomes of invasive aspergillosis (IA) are often unsatisfactory, the development of targeted immunotherapy remains an important goal. Linking the innate and adaptive immune system, dendritic cells are pivotal in anti-*Aspergillus* defense and have generated interest as a potential immunotherapeutic approach in IA. While monocyte-derived dendritic cells (moDCs) require *ex vivo* differentiation, antigen-pulsed primary myeloid dendritic cells (mDCs) may present a more immediate platform for immunotherapy. To that end, we compared the response patterns and cellular interactions of human primary mDCs and moDCs pulsed with an *A. fumigatus* lysate and two *A. fumigatus* proteins (CcpA and Shm2) in a serum-free, GMP-compliant medium. CcpA and Shm2 triggered significant upregulation of maturation markers in mDCs and, to a lesser extent, moDCs. Furthermore, both *A. fumigatus* proteins elicited the release of an array of key pro-inflammatory cytokines including TNF-α, IL-1β, IL-6, IL-8, and CCL3 from both DC populations. Compared to moDCs, CcpA- and Shm2-pulsed mDCs exhibited greater expression of MHC class II antigens and stimulated stronger proliferation and IFN-γ secretion from autologous CD4^+^ and CD8^+^ T-cells. Moreover, supernatants of CcpA- and Shm2-pulsed mDCs significantly enhanced the oxidative burst in allogeneic neutrophils co-cultured with *A. fumigatus* germ tubes. Taken together, our *in vitro* data suggest that *ex vivo* CcpA- and Shm2-pulsed primary mDCs have the potential to be developed into an immunotherapeutic approach to tackle IA.

## Introduction

*Aspergillus fumigatus* remains the most common mold pathogen causing life-threatening infections in immunocompromised patients such as hematopoietic stem cell transplant (HSCT) recipients. Mortality rates of invasive aspergillosis (IA) are up to 80%, mainly due to the limited efficacy of current diagnostic and therapeutic strategies. To meet the need for more specific therapeutic options, a spectrum of immunotherapeutic approaches have been developed to enhance host defence and support fungal clearance (1).

Dendritic cells (DCs) are an important cornerstone in the host response to *A. fumigatus* and have been proposed as a potential immunotherapeutic tool in IA. Highly phagocytic immature DCs patrol their environment and recognize pathogenic antigens *via* pattern recognition receptors (PRRs). Activated DCs present processed antigens to CD4^+^ and CD8^+^ T-cells and orchestrate the response of other innate and adaptive immune cell subsets through cytokine release and co-stimulatory surface receptors (2). *Ex vivo A. fumigatus* antigen-pulsed murine DCs have been shown to induce T_H_1 priming and confer protection against experimental IA (3). Similarly, DCs pulsed with live *A. fumigatus* or transfected with fungal RNA accelerated functional recovery of antifungal T_H_1 immunity in a murine HSCT model and had significantly greater therapeutic efficacy against murine IA compared to adoptive T-helper cell transfer (4).

Although distinct DC subsets differ in their potential to recognize fungal antigens *via* PRRs and display disparate phagocytic capacity, direct interaction studies revealed similar cytokine response patterns of monocyte-derived DCs (moDCs) and myeloid DCs (mDCs) upon confrontation with *A. fumigatus* morphotypes (5, 6). Of note, mDCs showed more pronounced MHC class II expression in response to *A. fumigatus* germ tubes than moDCs (5) and may therefore provide an appealing tool to augment anti-*Aspergillus* T-cell responses in immunocompromised patients. Despite their low abundance, mDCs can be readily isolated from whole blood or leukapheresate, whereas moDCs need to be induced through cytokine treatment of monocytes for a minimum of three days. As early therapeutic interventions are crucial to improve the outcome of IA (7), the immediate availability of mDCs may provide an important advantage for immunotherapeutic approaches. However, comparative studies of the functional properties of *ex vivo A. fumigatus* antigen-pulsed mDCs and moDCs and their interactions with other innate and adaptive immune cell subsets are scarce.

Moreover, the comparative stimulatory capacity of different classes of antigens in DCs is poorly understood. *Ex vivo* stimulation of DCs can be achieved with pathogen lysates, proteins, or peptides. Mycelial lysates contain complex and partially undefined components, such as cell wall proteins, glycostructures, lipids, and secondary metabolites, including immune-inhibitory metabolites (8). In contrast to lysates, a broad spectrum of proteins of a well-defined quality are available. Compared to individual peptides, proteins harbor a variety of antigens that can be presented by DCs to other immune cells, therefore initiating a more complex immune response than peptides. In this study, we compared *ex vivo* pulsing of mDC and moDC with an *Aspergillus* lysate and two proteins (CcpA and Shm2) that were recently identified as immunogenic *Aspergillus* antigens (9–11). We found that, in contrast to the lysate, these proteins reliably induce mDC-maturation and stimulate the release of pro-inflammatory cytokines and chemokines. Furthermore, antigen-pulsed mDCs elicited autologous T-cell proliferation, IFN-γ secretion, and enhanced the anti-*Aspergillus* oxidative burst of neutrophils. Importantly, protein-pulsed mDCs exhibited more pronounced maturation and greater T-cell stimulatory capacity than moDCs, suggesting the immunotherapeutic potential of mDCs in IA.

## Materials and Methods

### Aspergillus Antigens

*Aspergillus* proteins CcpA and Shm2 were recombinantly expressed as previously described (9). Briefly, the PCR-amplified open reading frame of *shm2* cDNA or a synthetic gene encoding for CcpA of *A. fumigatus* were amplified and cloned into the expression vector pET43.1H6 and pET28aH6TEV, respectively, for recombinant expression as His-tagged proteins. Proteins were purified by metal chelate affinity chromatography (Äkta explorer purification system, GE Healthcare). Protein concentrations were determined by measuring absorbance at 280 nm (NanoDrop 1000, Thermo Scientific) or by a Bradford protein assay (Biorad). Endotoxin contamination was determined (LAL Chromogenic Kit, Thermo Scientific) and reduced to <0.1 EU/ml using resin columns (Thermo Scientific). Research grade *A. fumigatus* lysate (AfuLy) was purchased from Miltenyi Biotec. To generate *A. fumigatus* germ tubes, 2×10^7^ conidia of strain ATCC 46645 were incubated in 20 mL RPMI, supplemented with 120 µg/mL gentamicin, for 9 h at 25°C in a shaking incubator (Infors HT, 200 rpm), pelleted at 5000 g for 10 min, and resuspended in RPMI + 5% FCS.

### Isolation and Generation of Dendritic Cell Subsets

Human peripheral blood mononuclear cells (PBMC) were obtained from leukocyte reduction chambers using Ficoll-Hypaque (Biochrome AG) density gradient centrifugation. Thereafter, mDCs were isolated from PBMCs by CD19 depletion followed by CD1c positive selection (Myeloid Dendritic Cell Isolation Kit, Miltenyi Biotec). Monocyte isolation from PBMCs was performed using MACS CD14 positive selection (Miltenyi Biotec). The moDCs were generated by incubation of monocytes with 20 ng/mL IL-4 and 100 ng/mL GM-CSF for 6 days as previously described (12). A minimum purity of ≥ 95% CD1c^+^ mDCs or CD14^-^ CD1a^+^ moDCs was confirmed by flow cytometry (FACS Calibur, Becton Dickinson, antibodies from Miltenyi Biotec). Furthermore, we confirmed at least 4-fold induction of the maturation markers CD80 and CD86 upon overnight stimulation with ultrapure LPS to validate the functionality of our DC preparations.

### Isolation of Polymorphonuclear Granulocytes (PMNs)

After obtaining informed consent, 15 mL venous EDTA blood was collected from healthy adult donors. PMNs were isolated using PolymorphPrep (Axis-Shield) gradient centrifugation according to the manufacturer’s instructions, except that Buffer EL (Qiagen) was used instead of Solution C. PMNs were suspended in RPMI 1640 + 10% FCS at a concentration of 3×10^6^/mL and the purity of the PMN suspension was verified by flow cytometry (CD3-PerCP^-^/CD66b-FITC^+^/CD14-PE^low^ cells > 90%, antibodies from Miltenyi Biotec).

### Flow Cytometric Analysis of DC Maturation

6×10^5^ mDCs or moDCs were cultured for 18 h at 37°C in 200 µL CellGro GMP DC medium (CellGenix, Cat. No.: 20801-0500) supplemented with 50 µg/mL AfuLy, 1-5 µg/mL *Aspergillus* proteins CcpA or Shm2, 1 µg/mL LPS (Sigma, positive control), or plain medium (negative control). Thereafter, DCs were stained with anti-CD1a-APC (moDCs) or anti-CD1c-APC (mDCs), anti-CD14-FITC, anti-CD80-APC, anti-CD83-PE, anti-CD86-FITC, anti-CD40-FITC, anti-HLA-ABC-PE, anti-HLA-DR-PE (BD), and anti-CCR7-APC (Miltenyi Biotec) in three different panels. Samples were analyzed using a FACS Calibur cytometer (BD), Cell Quest Pro software (BD), and Flow Jo software (version 10.6.2.). Dead cells were excluded from analysis by light scatter (FSC/SSC) properties.

### Analysis of Cytokine Release by Multiplex Cytokine Assays

6×10^5^ moDCs or mDCs were incubated for 18 h in plain CellGro medium (unstimulated control) or in 200 µL medium containing 50 µg/ml AfuLy, 5 µg/mL of *A. fumigatus* proteins CcpA or Shm2, 1 µg/mL ultrapure LPS (Invivogen), 100 µg/mL beta-glucan (Merck Millipore), or 6×10^5^ ethanol-inactivated *A. fumigatus* germ tubes. The supernatants were then collected and analyzed using a ProcartaPlex Human 8-plex plate assay (Thermo Scientific).

### IFN-γ ELISpot

Pulsing of moDCs and mDCs with antigens was performed for 18 h in 200 µL CellGro medium as described above. After pulsing, DCs were washed twice with CellGro to remove residual antigens. An ELISpot culture plate (Millipore) was washed 3 times with 200 µL PBS and coated with an IFN-γ capture antibody (1-D1K, BD), then incubated overnight at 4°C. The plate was then washed 5 times with PBS and blocked with CellGro medium for 3 h at 37°C. After removal of the medium, 5×10^5^ freshly isolated autologous T-cells (pan T-cell Isolation Kit, Miltenyi Biotec) were seeded in each well. Cells were stimulated with SEB + α-CD3 (1 µg/ml respectively, as a positive control) or 5×10^4^ autologous, *A. fumigatus* antigen-pulsed mDCs (T-cell-DC ratio 10:1). The ELISpot plate was incubated for 18 h at 37°C and subsequently washed 6 times with PBS. IFN-γ producing cells were stained for 2 h with an IFN-γ detection antibody (7-B6-1 biotin, BD) at RT, followed by another 5 PBS washing steps. After 1 h of incubation with a streptavidin-linked phosphatase (Southern Biotec) at RT, plates were washed 8 times with PBS and developed for 25 min with an NBT/BCIP substrate (Sigma). The staining process was stopped by rinsing the plate with water and spot counts were determined with an Immunospot S2A Core Analyzer ELISpot reader (CTL).

### T-Cell Proliferation Assay

The capacity of antigen-pulsed mDCs and moDCs to activate T-cell proliferation was evaluated by a carboxyfluorescein succinimidyl ester (CFSE)-based flow cytometry assay. DCs were pulsed with *Aspergillus* antigens for 18 h and washed twice with CellGro medium as described above. 5×10^4^ mDCs were co-cultured with 5×10^5^ autologous pan T-cells (1:10), stained with CFSE (5 µM, Life Technologies), in 550 µL CellGro medium for 6 days at 37°C. During this incubation, the remaining antigen-pulsed DCs were cryopreserved in 5CS-CryoStor medium (Stem Cell Technologies) at -80°C. Following restimulation with thawed antigen-pulsed DCs, T-cells were cultured for 6 additional days at 37°C and subsequently stained with fluorescently-labeled α-CD4 and α-CD8 antibodies (Miltenyi Biotec). The fraction of T-cells displaying reduced CFSE content compared to an unstimulated control was determined by flow cytometry.

### Stimulation of the Neutrophilic Oxidative Burst by DC Supernatants

6×10^5^ mDCs or moDCs were incubated in 200 µL CellGenix medium containing 50 µg/mL AfuLy, 5 µg/mL CcpA or 5 µg/mL Shm2, or in antigen-free medium for 18 h. Culture supernatants were sterile-filtered (0.2 µm) and cryopreserved at -80°C until further use.

In a 96-well plate, 1×10^5^ PMNs were combined with 5×10^4^ *A. fumigatus* ATTC 46645 germ tubes (in a total of 50 µL RPMI + 5% FCS) or were seeded in 50 µl RPMI + 5% FCS without addition of fungal cells. Thereafter, 33.3 µL of plain CellGenix medium or thawed DC supernatants diluted 1:4 with fresh CellGenix medium were added, containing the metabolites released by 2.5×10^4^ DCs (PMN-DC-ratio of 4:1). To quantify the release of reactive oxygen species, 16.7 µL dichlorofluorescein (DCF) solution was added (final concentration, 15 µg/mL). Thereafter, the plate was incubated for 2 hours in a temperature-controlled (37°C) SpectraMax iD3 Microplate Reader (Molecular Devices) and fluorescence was read every 5 min at an excitation and emission wavelength of 485 nm and 535 nm, respectively. Area-under-the-curve of time/relative fluorescence curves was calculated as a surrogate for the anti-*Aspergillus* oxidative burst. DC supernatants from three donors were combined with PMNs from three allogeneic donors for nine DC/PMN combinations. PMNs stimulated with phorbol 12-myristate 13-acetate (PMA, 10 ng/mL) were used as a positive control. PMN seeding density, DCF concentration, and multiplicity of infection were optimized in preceding experiments (unpublished data).

### Statistics

Significance testing was performed using the two-tailed, paired Student’s t-test or one-way repeated measures analysis of variance (RM ANOVA) with Dunnett’s post-hoc test, as detailed in the figure legends. Data were analyzed and visualized using Microsoft Excel and GraphPad Prism 8.

## Results

### CcpA and Shm2 Induce Time- and Concentration-Dependent mDC Maturation and Upregulation of Co-Stimulatory Molecules

**Figure 1:**
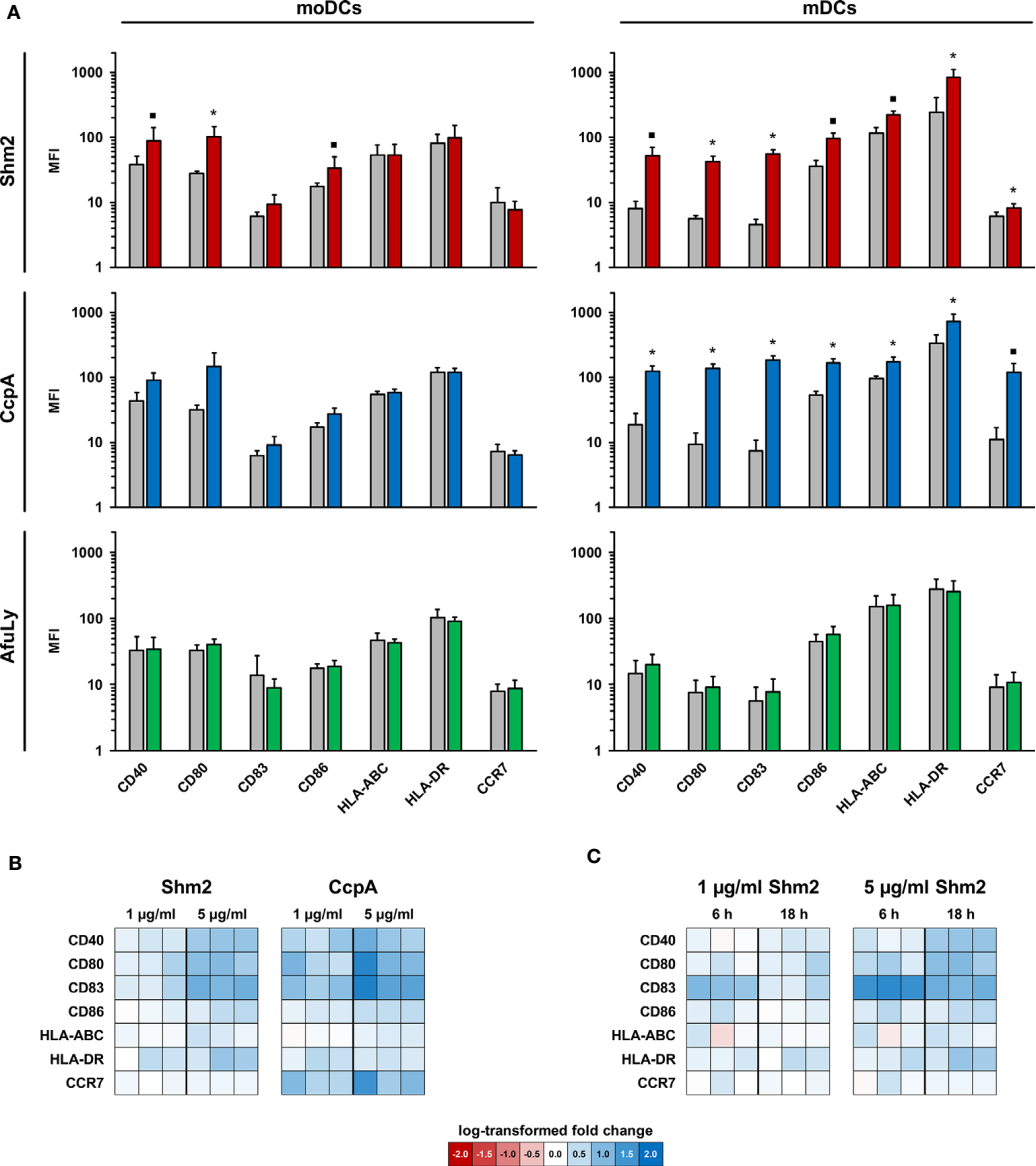
Maturation profiles of mDCs and moDCs stimulated with *A. fumigatus* antigens. **(A)** mDCs and moDCs were stimulated for 18 h with 5 µg/mL *Aspergillus* proteins Shm2 (red columns) or CcpA (blue columns), 50 µg/mL AfuLy (green columns), or plain medium (grey columns). Geometric mean fluorescence intensity values of surface maturation and activation markers were determined by flow cytometry. Means and standard deviations based on three independent donors are shown. The two-sided paired t-test was used for significance testing (stimulated versus unstimulated cells). ▪ p < 0.1, *p < 0.05. **(B, C)** Heat maps comparing activation marker expression in antigen-pulsed and unstimulated mDCs from three additional donors depending on the protein concentration **(B)** and incubation period **(C)**.

Maturation of DCs is necessary for efficient activation of adaptive immune cells (13). Therefore, we first assessed the ability of CcpA, Shm2, and AfuLy to induce MHC upregulation and maturation of mDCs and moDCs. After 18 h of stimulation, limited induction of CD40, CD80 and CD86 was found in moDCs stimulated with the proteinaceous antigens, whereas stimulation with the lysate did not alter surface expression of the maturation markers and co-stimulatory molecules that were tested (**Figure 1A**). While the lysate showed minimal stimulatory capacity in mDCs, Shm2 and CcpA induced greater upregulation (1.8-25.2-fold MFI) of all maturation markers tested compared with the unstimulated control (**Figure 1A**). CcpA led to a stronger induction of the co-stimulatory molecules CD80 and CD83 than Shm2 and elicited an 11-fold upregulation in the cell surface expression of homing receptor CCR7 on mDCs. For both CcpA and Shm2 stimulation, upregulation of maturation marker expression on the surface of mDCs was concentration-dependent (**Figure 1B**). For Shm2, we also evaluated the temporal kinetics of maturation marker expression by comparing mDCs exposed to 1 and 5 µg/mL Shm2 for 6 versus 18 hours. Except for the early activation marker CD83 (14), all differentially expressed markers showed more pronounced upregulation after overnight Shm2 stimulation (**Figure 1C**).

### Shm2 and CcpA Stimulate the Release of an Array of Pro-Inflammatory Cytokines From mDCs and moDCs

**Figure 2:**
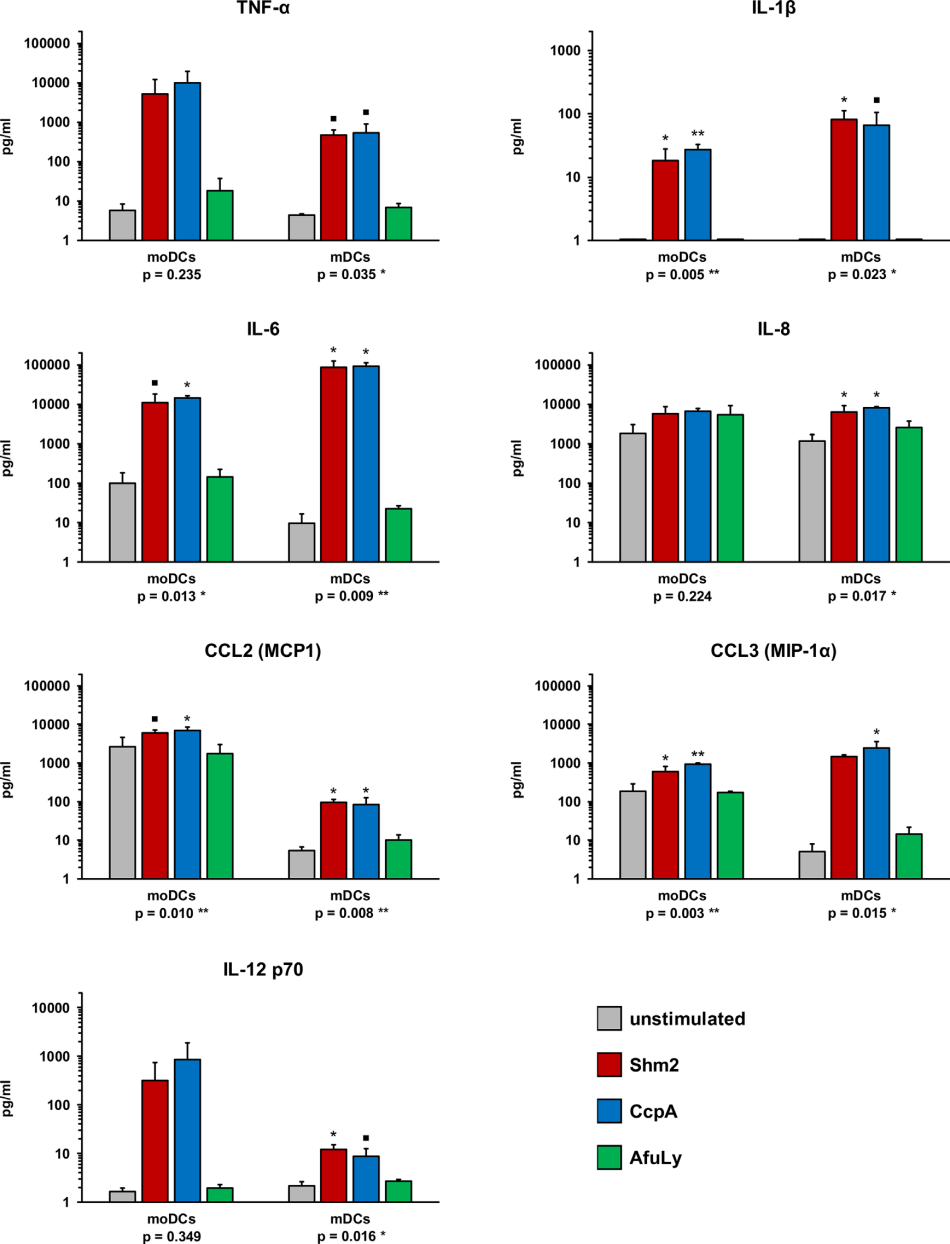
Cytokine profiles of *A. fumigatus* antigen-stimulated mDCs and moDCs. mDCs and moDCs were stimulated for 18 h with 5 µg/mL *Aspergillus* proteins Shm2 or CcpA, 50 µg/mL AfuLy, or plain medium (unstimulated). Cytokine concentrations in the supernatants were quantified using an 8-plex cytokine assay (data for IL-17A not shown, < 3 pg/mL for all conditions and donors). DCs from three independent donors were tested. Mean cytokine concentrations and standard deviations are shown. RM one-way ANOVA with Dunnett’s multiple comparison test (versus unstimulated cells). ▪ p < 0.1, *p < 0.05, **p < 0.01.

DCs secrete a broad spectrum of cytokines and chemokines orchestrating the response of other immune cell populations (15). Therefore, we stimulated mDCs and moDCs with Shm2, CcpA, and AfuLy for 18 h and compared cytokine concentrations in the culture supernatants with an 8-plex Luminex assay. Without stimulation, both DC subsets released consistently low amounts of TNF-α, IL-1β, and IL-12 p70, whereas baseline secretion > 10 pg/mL was seen for IL-6, CCL2 (MCP1), and CCL3 (MIP-1α) in moDCs and for IL-8 in both DC populations (**Figure 2**). AfuLy had no significant impact on cytokine release in either subset. Shm2 and CcpA strongly induced all tested cytokines (**Figure 2**) except IL-17A (data not shown) in both DC populations. While both protein antigens elicited higher levels of TNF-α (5.1-9.7 ng/mL) and IL-12 p70 (320-841 pg/mL) in moDCs than in mDCs, stronger induction of IL-1β (65-81 pg/mL), IL-6 (85-92 ng/mL), and CCL3 (1.5-2.4 ng/mL) was found in mDCs. Shm2 and CcpA induced greater absolute levels of CCL2 in moDCs than in mDCs, whereas relative induction compared to the unstimulated control was greater in mDCs.

Importantly, these minor quantitative differences in cytokine release by both DC subsets after Shm2 and CcpA stimulation were partially decoupled from cytokine responses elicited by PRR agonists (ultrapure LPS or beta-glucan) and inactivated *A. fumigatus* germ tubes (**Figure S1**). For example, higher concentrations of IL-6 and CCL3 (MIP-1α) were found in response to the PRR agonists and germ tubes, whereas Shm2 and CcpA induced stronger release of these cytokines by mDCs.

### Antigen-Pulsed mDCs and moDCs Stimulate IFN-γ Secretion and Proliferation of Autologous T-Cells

**Figure 3:**
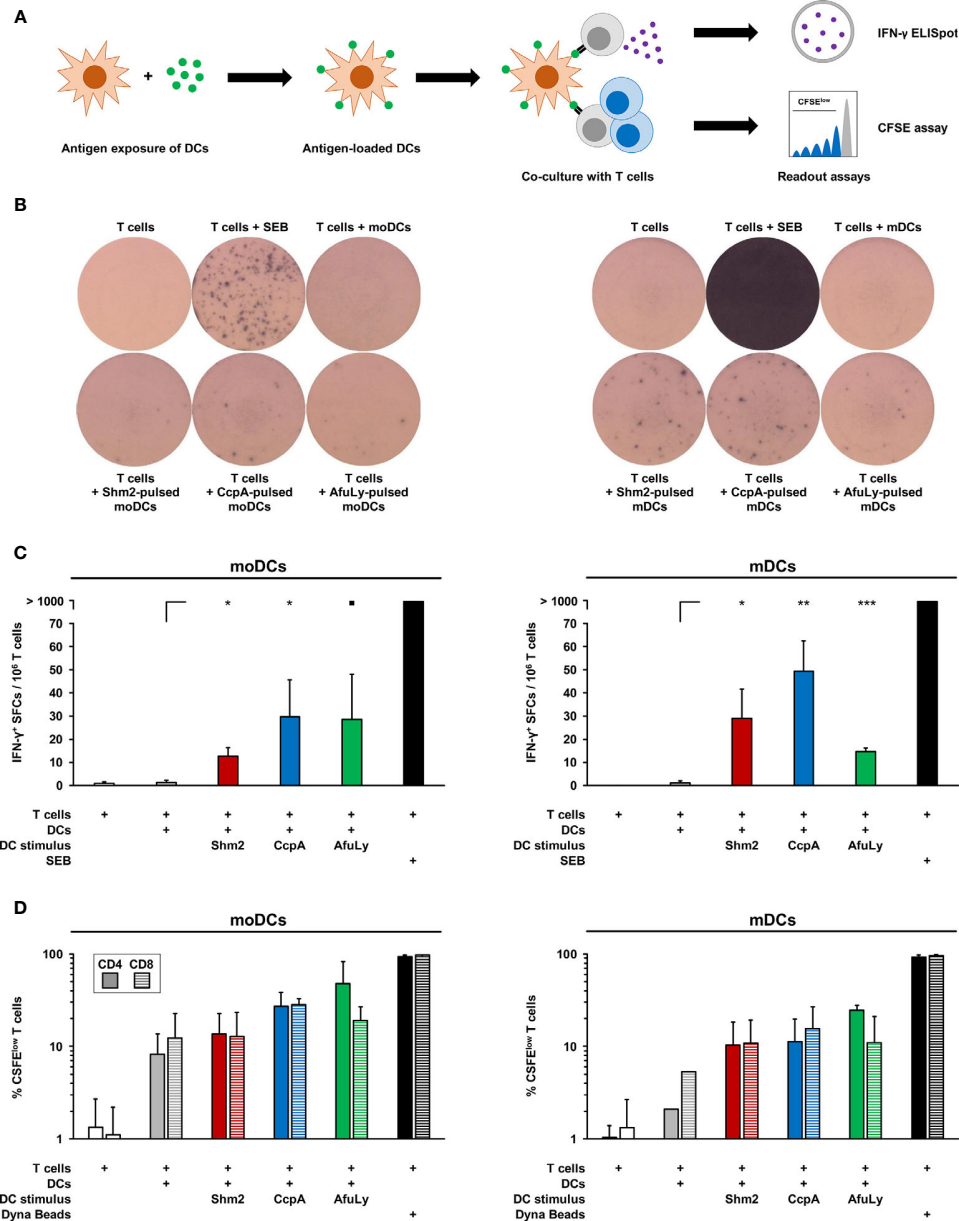
T-cell activation in co-cultures with antigen-loaded mDCs or moDCs. **(A)** mDCs and moDCs were loaded for 18 h with 5 µg/mL Shm2, 5 µg/mL CcpA, or 50 µg/mL AfuLy or kept in antigen-free medium (unstimulated). Subsequently, DCs were co-cultured with autologous T cells to quantify IFN-γ release by ELISpot and T-cell proliferation by a CFSE assay. **(B)** Images of ELISpot wells with cells from one representative donor are shown. **(C)** Mean spot forming cell (SFC) counts per 1×10^6^ T-cells and standard deviations are provided (n = 4). RM one-way ANOVA with Dunnett’s multiple comparison test (versus T-cells co-cultured with unstimulated DCs). ▪ p < 0.1, *p < 0.05, **p < 0.01, ***p < 0.001. **(D)** Frequencies of CFSE^low^ CD4^+^ and CD8^+^ T-cells in DC/T-cell co-cultures were measured by flow cytometry. As a proliferation control, T-cells were stimulated with Dynabeads CD3/CD28 T-cell Expander. Mean percentages of proliferating T-cells and standard deviations are shown (n = 2).

Next, we assessed the capacity of mDCs and moDCs pulsed with *Aspergillus* antigens to drive IFN-γ secretion from autologous T-cells, a cytokine that is implicated in protective immunity to fungal infections (16–18). Therefore, DCs were stimulated for 18 h with Shm2, CcpA, or AfuLy, washed, and co-cultured with autologous T-cells in an IFN-γ ELISpot plate (**Figure 3A**). Compared to unstimulated mDCs, pulsing with CcpA, Shm2, and AfuLy caused significant induction of IFN-γ secretion by autologous T-cells (**Figures 3B, C** **)**. Interestingly, despite less pronounced upregulation of maturation markers compared with mDCs (**Figure 1A**), Shm2- and CcpA-pulsed moDCs also stimulated T-cells to release IFN-γ (**Figures 3B, C** **)**. For AfuLy, an even stronger response was seen in moDC- than in mDC-co-cultures (**Figure 3C**). However, the strongest IFN-γ response was elicited by CcpA-pulsed mDCs (**Figure 3C**). Importantly, no direct induction of IFN-γ spot formation was seen in T-cell monocultures stimulated with the antigens (data not shown), precluding that spot formation in co-cultures was due to unprocessed antigens remaining in the medium after harvesting and washing of DCs.

To corroborate that antigen-pulsed mDCs induce autologous T-cell activation, we quantified the frequencies of proliferating CD4^+^ and CD8^+^ cells with a flow cytometric CFSE-based assay (**Figure 3A**). T-cells alone or T-cells co-cultured with unstimulated mDCs showed low proliferation rates of 1.0-5.2%, whereas mDCs pulsed with Shm2, CcpA, and AfuLy induced significant proliferation of CD4^+^ T-helper cells (10.3-24.6%) and cytotoxic CD8^+^ T-cells (10.9-15.5%, **Figure 3D**). While unstimulated moDCs induced stronger T-cell proliferation than mDCs, antigen loading of mDCs elicited greater relative changes in T-cell proliferation, particularly for CD8^+^ T-cells.

### Supernatants of Shm2- and CcpA-Pulsed mDCs Enhance the *Aspergillus*-Induced ROS Release of PMNs

**Figure 4:**
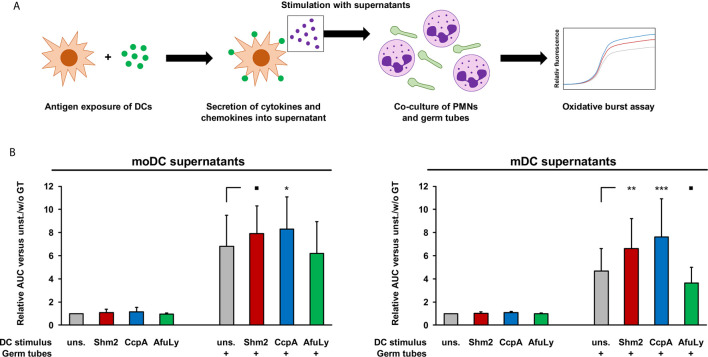
Stimulatory activity of DC supernatants on *A. fumigatus*-induced oxidative burst in allogeneic PMNs. **(A)** mDCs and moDCs were loaded for 18 h with 5 µg/mL Shm2, 5 µg/mL CcpA, or 50 µg/mL AfuLy or kept in antigen-free medium (unstimulated). Supernatants were collected and cryopreserved. Allogeneic PMNs (1×10^5^/well) were co-cultured with *A. fumigatus* germ tubes (5×10^4^/well) in medium supplemented with diluted DC supernatants, with the medium of each well containing the metabolites from 2.5×10^4^ DCs. ROS release was quantified using a DCF-based assay. **(B)** Two-hour AUC values of relative fluorescence/time curves were determined and normalized to unstimulated PMNs incubated in medium containing supernatants from unstimulated DCs. Mean relative AUCs based on 9 PMN/DC combinations (3 DC donors × 3 PMN donors) and standard deviations are shown. RM one-way ANOVA with Dunnett’s multiple comparison test (versus PMN/germ tube co-cultures with unstimulated DC supernatants). ▪ p < 0.1, *p < 0.05, **p < 0.01, ***p < 0.001.

In addition to their impact on T-cell activation and proliferation, we compared the capacity of supernatants from antigen-pulsed mDCs and moDCs to enhance the anti-*Aspergillus* oxidative burst of PMNs (**Figure 4A**). Regardless of antigen pulsing, baseline ROS secretion of unstimulated PMNs in the presence of DC supernatants remained consistently low (fold change, 0.94-1.16, **Figure 4B**). With germ tube stimulation, we observed at least a 3.6-fold increase in ROS release after 2-hours with all supernatants. Although unstimulated moDC supernatants caused a greater neutrophilic ROS response to the germ tubes than unstimulated mDC supernatants, a stronger relative increase in ROS release was observed with supernatants from mDC when pulsed with protein antigens, especially with CcpA (+62%, **Figure 4B**, right panel). In contrast, pulsing of DCs with the lysate reduced the stimulatory capacity of their supernatants on the neutrophilic ROS response.

## Discussion

Dendritic cells are pivotal in mounting an innate and adaptive immune response against *A. fumigatus* and are considered a potential tool for anti-*Aspergillus* immunotherapy (19, 20). Because of their ability to present antigens to initiate the generation of antigen-specific CD4^+^ and CD8^+^ T-cell responses, *ex vivo*-generated DCs have been applied in antiviral and antitumor vaccine strategies (21–23). One of the few clinical studies to have exploited primary CD1c^+^ mDCs used them for the treatment of metastatic melanoma (24). These authors showed that therapeutic vaccination with less than 10^7^ mDCs is feasible and without substantial toxicity. Four of 14 patients showed long-term progression-free survival (12-35 months), which directly correlated with the development of multifunctional CD8^+^ T-cell responses in three of these patients (24). So far, to our knowledge, no study exists on the use of human mDCs for anti-*Aspergillus* immunotherapy.

In this study, we propose two *Aspergillus* proteins, CcpA and Shm2, as potential candidates for the development of a dendritic cell based anti-*Aspergillus* immunotherapy. CcpA is a fungus-specific, 25.5 kDa amphiphilic protein highly abundant in the conidial cell wall but disappearing during the germination process. CcpA is required for fungal virulence in immunocompromised mice with IPA, positioning the protein as a potential target for immunotherapy (11). Shm2 is a ubiquitous, 51.9 kDa protein found in all pro- and eukaryotes, and plays a central role in providing one-carbon units for biosynthetic processes (25). Shm2 is present on the surface of *A. fumigatus* germinating conidia and hyphae (26), was found to trigger IgG responses in sera of patients with invasive aspergillosis (9), and is a strong elicitor of memory T cell responses (9).

Interestingly, our data revealed higher maturation levels of mDCs pulsed with CcpA than Shm2 and greater IFN-γ secretion of autologous T cells co-cultured with CcpA-pulsed mDCs. Moreover, stronger enhancement of T-cell proliferation was observed upon co-culture with CcpA-pulsed mDCs than Shm2-pulsed mDCs. Furthermore, compared with Shm2, CcpA induced strong expression of the homing receptor CCR7 in *ex vivo* mDCs, suggesting that CcpA-pulsed mDCs are able to migrate to the lymph nodes and activate an antigen specific T-cell response (27, 28).

*A. fumigatus* belongs to the group of pathogens whose antigens are predominantly presented *via* class II MHC molecules. Accordingly, strong proliferation of MHC class II-restricted CD4^+^ T-helper cells was found upon stimulation by mDCs pulsed with CcpA or Shm2. However, we also observed considerable proliferation of MHC class I-restricted CD8^+^ cytotoxic T cells, which may be explained by cross presentation. For cross presentation, extracellular antigens are internalized into the cytosol, processed, and loaded on MHC class I molecules (29). Interestingly, unlike bacteria and viruses, fungi can elicit long-term CD8^+^ T cell memory that is maintained without CD4^+^ T cell help or persistent antigen (30). In agreement with this hypothesis, both *A. fumigatus* proteins moderately induced class I MHC expression on mDCs.

The mycelial lysate evoked neither significant maturation of mDCs nor meaningful cytokine secretion. This observation may be due to the presence of metabolites which inhibit DC activation. As an example, Stanzani and colleagues described that gliotoxin in cell wall lysates of *A. fumigatus* has an immunosuppressive effect that specifically results in functional impairment of moDCs (8). In addition, galactosaminogalactan, also present in *A. fumigatus* lysates, exerts immunomodulatory effects through the modulation of cytokine production, e.g., by modulating the IL-1 signaling pathway that is crucial for DC activation (31, 32). Furthermore, the commercial lysate used in this study might contain various unknown additives, such as stabilizers and preservatives, which could additionally inhibit DCs (and neutrophils). Nonetheless, T cells co-cultured with *A. fumigatus* lysate-pulsed mDCs released IFN-γ and proliferated. If high affinity compounds from the T-cell-optimized lysate are directly loaded to surface MHC molecules, T cells may be stimulated by lysate-pulsed mDCs without induction of significant mDC maturation and cytokine secretion (13). Considering such lysates for *ex vivo* DC pulsing, co-stimulation with adjuvants may be warranted to elicit optimal maturation marker induction and cytokine secretion (33, 34).

Our study revealed important data about the *in vitro* interplay between human primary mDCs and *A. fumigatus* antigens focusing on molecular and functional mechanisms, which in future might allow the development of immunomodulators for more effective antifungal immunotherapy. This includes additional *in vitro* and *in vivo* studies on the potential use of appropriate adjuvants for mDC activation, such as TLR ligands (e.g., Pam3CDK4 or CpG-containing oligonucleotides) (35), the combined use of mDCs and autologous pDCs (36), or the exploitation of the potential immunomodulatory effects of antifungals, especially echinocandins, on *ex vivo*-pulsed mDCs. Although a GMP-grade medium was used for DC culture and stimulation in this study, further standardization of the antigens and protocols for future GMP-compliant products will be needed. In addition, further studies are required using mammalian models to evaluate the persistence of *ex vivo*-stimulated mDCs *in vivo*, their capacity to migrate to the lymph nodes, as well as their safety and potential immunogenic potential. Future *in vivo* studies should also include direct comparisons of the feasibility, safety, and efficacy of *ex vivo*-pulsed mDCs with other potentially promising cell therapeutics (e.g., adoptive T-cell transfer or mold-reactive CAR T-cells) (4, 37, 38) and non-cellular immune enhancement strategies such as immune checkpoint blockade (39).

Despite these limitations and need for further research, the observed broad pro-inflammatory cytokine spectrum, upregulation of co-stimulatory molecules and MHC complexes, as well as subsequent activation of T-cell proliferation, and neutrophilic oxidative burst, position mDCs as a potential tool for anti-*Aspergillus* immunotherapy. Furthermore, our findings suggest that Shm2, and particularly CcpA, are promising candidates for the development of an antigen cocktail to obtain functional *ex vivo* mDCs for adoptive transfer approaches.

## Data Availability Statement

The raw data supporting the conclusions of this article will be made available by the authors, without undue reservation.

## Ethics Statement

The studies involving human participants were reviewed and approved by Ethics Committee of the University Hospital of Wuerzburg (protocol number #302/12). Written informed consent for participation was not required for this study in accordance with the national legislation and the institutional requirements.

## Author Contributions

LP and JW performed experiments and contributed to data analysis and manuscript preparation. JLot, MB and LS performed experiments. OK, VV, JT, and PH provided key reagents and contributed to experimental procedures. CM contributed to the study design and manuscript preparation. AB, MT, and HE contributed to the study design and supervision. SW contributed to the study design and supervision, analyzed and visualized the data, and wrote the manuscript. JLoe conceived and supervised the study and wrote the manuscript. All authors contributed to the article and approved the submitted version.

## Funding

The study was supported by the Deutsche Forschungsgemeinschaft (DFG) within the Collaborative Research Center CRC124 FungiNet “Pathogenic fungi and their human host: Networks of interaction”, DFG project number 210879364 (projects A1 to AB, A2 to HE and JLoe, A4 to MT, Z2 to OK, and travel support to CM and SW). We would like to thank the Department of Transfusion Medicine of the University Hospital of Wuerzburg for providing leukocyte reduction systems for PBMC isolation, and both Lydia Bussemer and Sylke Fricke for technical support.

## Conflict of Interest

The authors declare that the research was conducted in the absence of any commercial or financial relationships that could be construed as a potential conflict of interest.

## Supplementary Material

Supplementary Figure 1 Cytokine responses of mDCs and moDCs elicited by PRR agonists and inactivated *A. fumigatus* germ tubes. mDCs and moDCs were stimulated for 18 h with 1 µg/mL ultrapure LPS (TLR4 agonist), 100 µg/ml beta-glucan (Dectin-1 agonist), inactivated *A. fumigatus* germ tubes (multiplicity of infection = 1.0), or plain medium (unstimulated). Cytokine concentrations in the supernatants were quantified using an 8-plex cytokine assay (data for IL-17A not shown, < 20 pg/mL for all conditions and donors). DCs from three independent donors were tested. Mean cytokine concentrations and standard deviations are shown. RM one-way ANOVA with Dunnett’s multiple comparison test (versus unstimulated cells). ▪ p < 0.1, *p < 0.05, **p < 0.01.
